# Validating nutrient selection for product-group-specific nutrient indices for use as functional units in life cycle assessment of foods

**DOI:** 10.1017/S0007114524000709

**Published:** 2024-06-28

**Authors:** Anna Kårlund, Venla Kyttä, Tiina Pellinen, Hanna L. Tuomisto, Anne-Maria Pajari, Marjukka Kolehmainen, Merja Saarinen

**Affiliations:** 1 Institute of Public Health and Clinical Nutrition, University of Eastern Finland, Kuopio, Finland; 2 Department of Life Technologies, University of Turku, Turku, Finland; 3 Natural Resources Institute Finland (Luke), Helsinki, Finland; 4 Department of Food and Nutrition, University of Helsinki, Helsinki, Finland; 5 Department of Agricultural Sciences, University of Helsinki, Helsinki, Finland; 6 Helsinki Institute of Sustainability Science, University of Helsinki, Helsinki, Finland

**Keywords:** LCA, Nutrient index, nFU, Validation, Principal component analysis

## Abstract

The ability to provide adequate nutrition is considered a key factor in evaluating the sustainability of foods and diets. Nutrient indices are used as functional units (FU) in life cycle assessment of foods to include nutritional performance in the environmental assessment of a product. Several general and food-group-specific nutrient indices exist but many lack validation, particularly when used as FU. In addition, the nutrient selection strategies and reference units for nutrient intake can vary considerably among studies. To validate intake-based product-group-specific nutrient indices previously developed for protein (NR-FI_prot_) and carbohydrate (NR-FI_carb_) foods and for fruits and vegetables (NR-FI_veg_), we applied principal component analysis to investigate correlations between nutrients in foods and dishes representing a typical Finnish diet. The reference amounts for meal components were based on a plate model that reflected Finnish dietary recommendations. The portion sizes for the different food groups were anchored at 100 g, 135 g and 350 g for proteins, carbohydrates and fruits/vegetables, respectively. Statistical modelling largely validated the NR-FI indices, highlighting protein foods as sources of niacin, vitamin B_12_ and Se, carbohydrate foods as sources of Mg, Fe and phosphorous, and fruits/vegetables as sources of potassium, vitamin K, vitamin C, fibre and thiamine. However, in contrast to the intake-based approach applied in NR-FI_prot_, the dietary recommendation-based validation process suggested that fruits and vegetables should be favoured as sources of riboflavin and vitamin B_6_.

Nutritional quality has recently been recognised as a fundamental sustainability issue^(1)^. In life cycle assessment (LCA), this has led to the development of nutrient indices that can be used as nutritional functional units (nFU) in LCA for foods. FU are established to compare the environmental performance of products for their intended purpose^(2)^; for foods, this refers to their ability to provide nutrients. Different types of nFU can be applied, for example, they can be based on a single nutrient or a nutrient profiling score^(3)^. The Nutrient Rich Food (NRF) index^(4)^ and its variants^(5)^ are among the most used nutrient indices used as nFU in LCA of food products. In addition, several product-group-specific indices have been developed to enable more precise evaluation of environmental impacts per nutrients provided, highlighting the role of protein sources, a product group with both a specific nutrient profile and potentially high environmental footprint^(5–7)^. Product-group-specific methods are based on the idea that foods in a product group can be substituted with other products in the same group^(8)^. Thus, the principle is slightly different from the across-the-board indices that are aimed to provide guidance, which foods to include in or exclude from a diet based on their healthiness or unhealthiness, respectively^(8)^. Product-group-specific indices used as nFU in LCA include, for example, NRF_protein-sub_^(9)^, NQI^(6)^, FNI_prot_7^(7)^ and its variations^(3)^ for protein and Nu index for carbohydrate-rich foods^(10)^.

Nutrient indices indicate the amount of selected nutrients that are provided by foods in relation to the given nutrient-specific intake recommendations. A nutrient index can be built to indicate the presence of generally beneficial nutrients, to highlight adequate intake of critical nutrients for which there is a risk of deficiency, and/or to encourage adherence to specific dietary guidelines. Public health perspectives and national dietary guidelines have been considered especially important for nutrient selection strategies^(11)^. In this regard, nutrients that should be limited, such as SFA, sugar and/or Na, are sometimes included in the index. However, this can lead to negative index values and thus disable their application as nFU in LCA^(12)^.

Establishing the evaluation of nutrient intake from a food product on product-specific portion size (instead of, e.g. 100 g or 100 kcal) has been considered understandable for consumers and able to highlight healthy food choices^(11)^. As a framework for a balanced meal, the National Nutrition Council of Finland introduced the plate model, which recommends 1/4 of plate for protein, 1/4 for carbohydrate and 1/2 for vegetables, plus one slice of bread and one portion of berries for healthy adults^(13)^. The model is familiar to consumers, and the ratios of different food groups can be fine-tuned for the needs of different population groups. Thus, the components of the plate model can be used as a starting point for the portion sizes and also for product grouping, indicating foods that have corresponding roles in a meal or a diet. Based on this principle, we have recently introduced product-group-specific nutrient indices to be used as nFU in LCA for protein sources (NR-FI_prot_)^(14)^, vegetables, fruits and berries (NR-FI_veg_), and carbohydrate sources (NR-FI_carb_)^(15)^. The reference unit for these indices was standardised at 100 g, and the nutrients were selected for the indices based on the national intake data from Finland^(16)^ to represent macro- and micronutrients for which the product group in question serves as a major source.

A more harmonised way of selecting nutrients for nutrient indices is needed^(11,17)^, and one of the identified areas of future research on nutrient indices as FU is the validation process for the indices^(12)^. Nutrient profile models, that is, ranking of foods from healthy to unhealthy based on their nutrient compositions, have been validated against nutrition professional surveys^(18)^ and recommendations^(19)^ and measures of diet quality, such as the Healthy Eating Index (HEI)^(4)^. In the case of product-group-specific indices, unsupervised statistical methods could be used for validation purposes to support expert knowledge to ensure that a nutrient index truly reflects the nutritional function suggested for a product group. These methods allow analysis of patterns in nutrient composition data without assuming any grouping of variables in advance. Statistical methods have been used also before in an attempt to establish relationships between nutritional benefits and environmental sustainability^(20)^ and to evaluate across-the-board index performance against HEI^(4)^, for example. However, very few previous studies have utilised statistical methods to select nutrients for nutrient indices and to validate food grouping for product-group-specific indices.

In this study, our objective was to validate how well the recently developed NR-FI indices represent the nutritional functions of the different product groups and if the suggested product grouping corresponds to these functions. For this purpose, we used a statistical dimension reduction method to identify which nutrients are correlated in foods that are typically consumed in the everyday Finnish diet. The plate model is used to estimate the portion sizes of different product groups for a healthy adult. We aim to contribute to the discussion on the strengths and limitations of different strategies to select nutrients for indices that can be used as FU in LCA.

## Materials and methods

### Food data

**Fig. 1.**
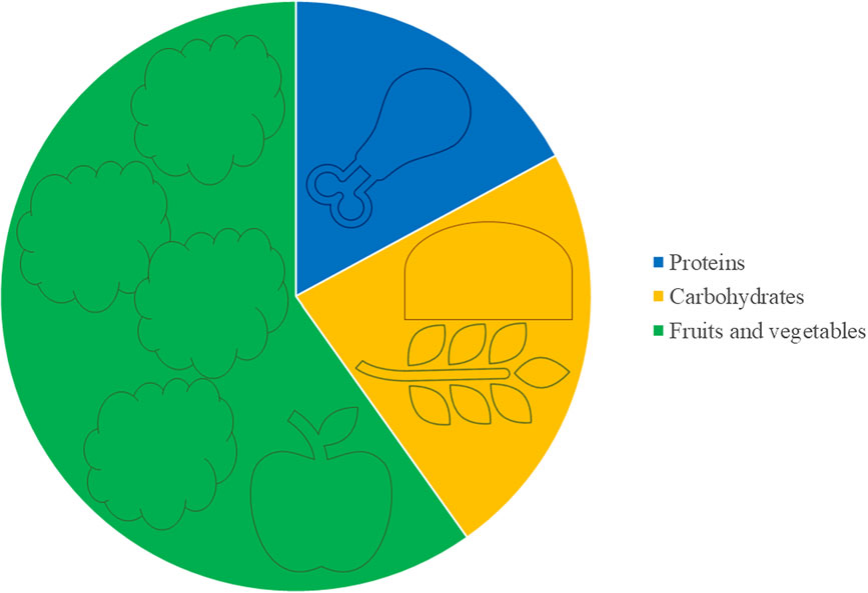
The plate model used to anchor the portion sizes to determine the intake of nutrients from different food groups. The portion sizes were 100 g, 135 g and 350 g for protein source, carbohydrate source and fruits and vegetables, respectively.

Example foods and their nutrient compositions were collected from the national Food Composition Database in Finland (Fineli)^(21)^. The foods were selected to represent typical main and side dishes, for example, in home cooking and lunch cafeterias, excluding mixed foods, such as soups and casseroles, combining both carbohydrates and proteins in a single dish. In addition, we included common fresh and cooked fruits and vegetables in the dataset. The complete list of foods is presented in online Supplementary Table S1. The original set of foods contained seventy-two food items, including twenty-four protein dishes (eight red meat (i.e. three beef, two pork, one reindeer, one frankfurter and one liver), six plant-based, five fish, four poultry and one egg), twenty-four carbohydrate foods (seven grains, five tubers, four breads, four pastas, three morning cereals and one bun), and twenty-four fruits and vegetables (seven salad mixes, six fruits, five berries, five raw/cooked vegetables and one berry kissel). The portion sizes that were used to determine the total nutrient contents were selected based on a commonly adopted plate model containing 1/4 protein, 1/4 carbohydrate and 1/2 vegetables^(13,22,23)^. In addition, one small to large portion of fruit/berries and a slice of bread were considered as part of the model meal (Fig. 1)^(13)^. The final portion sizes were anchored as follows: 100 g for protein foods, 135 g for carbohydrate foods and 350 g for fruits/vegetables. On average, the model meal contained 535 kcal.

### Statistical analysis

Clustering of nutrients in food items was analysed using principal component analysis (PCA; SPSS Statistics software version 26, IBP corp.) with varimax rotation. Nutrient contents (online Supplementary Table S1) were used as variables. In PCA, the principal components represent newly created, uncorrelated variables that are linear functions of the variables in the nutrient composition data, whereas rotation aims to maximise the contribution of interrelated nutrients in one component while minimising their contribution to the other components^(24,25)^. Components were first extracted based on eigenvalue threshold 1^(24)^; this resulted in seven components, explaining 79·6 % of the variance. After observing the plateauing of the eigenvalue diagram (i.e. scree plot indicating the most significant components explaining the variance) and the number of variables loaded on each component (to eliminate components with only 1–2 nutrients), the number of components was reduced to five. Because nutrients that were at high levels in minced liver steak or in carbohydrate sources containing milk (bun, multigrain bread and rice porridge) were driving separation of specific components (online Supplementary Table S2), these foods were removed from the dataset as anomalies of their corresponding product groups. Berry kissel was also removed due to very low content of any nutrients. The final list of food items is presented in Table 1. After food item exclusion, another PCA with eigenvalue threshold 1 was conducted. Based on the scree plot, components were again reduced and the final rotated component matrix contained three components. SIMCA® (Multivariate Data Analysis software version 16, Umetrics) was used to build score plots and loading plots for product types and nutrients, respectively. A heat map showing which food items were highest and which were lowest in the nutrients that were clustered in the PCA was prepared using Excel (Microsoft corp.). Food items were colour-coded based on the food group (protein, carbohydrate and fruit/vegetable), and for each nutrient, food items were sorted for their content from highest to lowest and grouped based on the three PCs (PC1, PC2 and PC3). Vitamin A was excluded from the heat map for having a loading < 0·3 in the PCA.

**Table 1.** Food items included in the validation process

Protein foods		Carbohydrate foods		Fruits and vegetables	
Code	Food	Code	Food	Code	Food
Eggs		Pasta		Greens	
E1	Scrambled eggs, low-fat milk	A1	Macaroni, boiled without salt	G1	Aubergine
Fish		A2	Macaroni, dark, boiled without salt	G2	Beetroot, boiled without salt
F1	Baltic herring, breaded, fried	A3	Oat macaroni, boiled, no added salt	G3	Broccoli, boiled without salt
F2	Fish fingers, oven-baked, industrial	A4	Rye macaroni, boiled, without salt	G4	Carrot
F3	Saithe with white sauce, cheese, low-fat milk, breadcrumbs, oven-baked	Bread		G5	Swede, boiled without salt
F4	Salmon cake, fried	B2	Rye bread, water, wholegrain rye flour	Salads	
F5	Salmon, warm smoked	B3	Toast, multigrain toast	S1	Salad, cabbage and lingonberry
Meat		B4	White bread, wheat bread, French bread, industrial	S2	Salad, cabbage, cucumber and leek
M1	Beef mince patty, fried	Breakfast cereal		S3	Salad, Chinese cabbage, pea, sweet corn and sweet pepper
M2	Chili con carne	C1	Breakfast cereal, Rice Krispies	S4	Salad, iceberg lettuce, carrot and zucchini
M3	Frankfurter gravy	C2	Muesli with dried fruit and nuts	S5	Salad, lettuce, cucumber and tomato
M4	Karelian stew, pork and beef, root vegetables	C3	Oat porridge, oat flakes, water, without salt	S6	Salad, swede and pineapple, grated
M6	Pork fillet, sirloin, oven-baked	Grains		S7	Salad, tomato, bean and onion
M7	Pork-vegetable wok, wok-vegetables	R1	Buckwheat, boiled without salt	Fruit	
M8	Sautéed reindeer, low-fat	R2	Quinoa, cooked without salt	U1	Apple, domestic, with skin
Poultry		R3	Rice and oat mix, boiled, without salt	U2	Banana, without skin
P1	Chicken fricassee	R5	Rice, brown, boiled without salt	U3	Grape, average, green or red
P2	Chicken leg and thigh without skin, oven-baked	R6	Rice, long-grain, boiled without salt	U4	Watermelon, without skin
P3	Chicken-vegetable wok, wok-vegetables	R7	Rice, quinoa and multigrain mix, boiled, without salt	U5	Orange, without skin
P4	Turkey mince patty, fried	Tubers		U6	Peach/nectarine, average, with stone
Plant protein		T1	French fries, oven-baked	Berries	
V1	Bean quinoa patty, fried	T2	Mashed potatoes, water, cooking fat	Y1	Blackcurrant
V2	Bean stew, water, dried beans	T3	Potato, baked with skin, no filling	Y2	Blueberry, bilberry *Vaccinium myrtillus*
V3	Vegetable wok with minced faba bean, wok-vegetables	T4	Potato, peeled, boiled without salt	Y3	Raspberry
V4	Soy Bolognese	T6	Sweet potato fries, deep-fried	Y4	Sea buckthorn berry
V5	Tofu vegetable wok, wok-vegetables			Y5	Strawberry
V6	Tofu, breaded, fried in oil				

### Comparison with NR-FI indices

The nutrients associated with each component were compared with the nutrients included in the NR-FI indices^(14,15)^. The baseline index for protein-rich foods NR-FI_prot_ includes protein, Ca, Fe, Se, Zn, vitamins B_6_ and B_12_, niacin, riboflavin and thiamine, the baseline index for sources of carbohydrates NR-FI_carb_ index includes carbohydrates, fibre, Fe, Mg, phosphorous, potassium and folate, and the baseline index for vegetables, fruits and berries NR-FI_veg_ index includes fibre, potassium, thiamine, and vitamins C, K and A.

The NR-FI indices were calculated with formula:

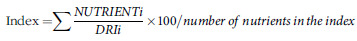

where *NUTRIENTi* is the amount of a nutrient in 100 g of a product and *DRIi* is the daily recommended intake of *NUTRIENTi* in the nutrition and food recommendations of The National Nutrition Council of Finland^(26)^. The nutrient index scores were calculated for men and women aged 10–13, 14–17, 18–30, 31–60, 61–74, and over 75 years, as well as for children aged 12–23 months, 2–5 years, and 6–9 years^(14,15)^.

## Results

### Nutrient clustering in the principal component analysis

The three components that were formed in the PCA explained 53·7 % of the variance among the foods (Table 2). PC1 was positively associated with protein, niacin, vitamin B_12_, MUFA, iodine, vitamin D, PUFA and Se. PC2 correlated positively with potassium, vitamin K, riboflavin, vitamin C, folate, fibre, Ca, vitamin E, thiamine and vitamin B_6_. Mg, Fe, phosphorus, Zn and available carbohydrates were positively loaded on PC3.

**Table 2.** Rotated component matrix from the principal component analysis (PCA) on nutrient contents of foods^(21)^ that were included in the validation process of NR-FI indices^(14,15)^

	Component		
	1	2	3
Variance explained, %	23·6	20·4	9·7
Protein (g)	**0·861**		
Niacin (mg)	**0·770**		
Vitamin B_12_ (µg)	**0·755**		
MUFA (g)*	0·752		
Iodine (µg)*	0·720		
Vitamin D (µg)*	0·711		
PUFA (g)*	0·704		
Se (µg)	**0·589**	0·471	
Potassium (mg)		**0·808**	†
Vitamin K (µg)		**0·737**	
Riboflavin (mg)	*0·345*	0·721	
Vitamin C (mg)		**0·695**	
Folate (µg)		0·682	†
Fibre (g)	–0·389	**0·676**	*0·393*
Ca (mg)	†	0·483	
Vitamin E (mg)*		0·482	
Thiamine (mg)	†	**0·446**	
Vitamin B_6_ (mg)	†	0·401	
Vitamin A (µg)		**0·279** ‡	
Mg (mg)			**0·830**
Fe (mg)	†		**0·804**
Phosphorus (mg)	0·538		**0·718**
Zn (mg)	*0·538*		0·598
Carbohydrate, available (g)	–0·405		**0·502**

Loadings < 0·3 are not included in the table. Nutrients that were both loaded on the same component and corresponding with the same NR-FI index are in bold. PC1 correlated with four nutrients included in NR-FI_prot_ index (protein, niacin, vitamin B_12_ and Se), PC2 correlated with five nutrients included in NR-FI_veg_ index (K, vitamin K, vitamin C, fibre and thiamine) and PC3 correlated with four nutrients that were included in NR-FI_carb_ index (Mg, Fe, P and available carbohydrate). Nutrients that are not primarily loaded on the component that cluster their corresponding NR-FI index-specific nutrients but show secondary association are italicised.

* Not included in any NR-FI baseline index.

† Not validated to associate with corresponding NR-FI baseline index.

‡ Loading < 0·3.

Riboflavin and fibre, as well as phosphorous and Zn, showed weaker positive association with another PC in addition to PC2 and PC3, respectively. Riboflavin, phosphorous and Zn showed correlation with PC1 and fibre with PC3.

### Correspondence to NR-FI indices

Nutrients that are associated with each other in NR-FI indices, as well as in the PCA of the current study, are indicated in Table 2. In correspondence to NR-FI_prot_, the PCA was able to associate protein with niacin, vitamin B_12_ and Se. In addition, riboflavin and Zn showed secondary association with these nutrients. As in NR-FI_carb_, carbohydrate was associated with Mg, Fe and phosphorous, and at a secondary level with fibre. Several nutrients that were included in NR-FI_veg_ were associated with PC2. These nutrients included potassium, vitamin K, vitamin C, fibre and thiamine. Furthermore, although vitamin A, which was included in NR-FI_veg_, did not quite exceed the threshold that was set for qualification of loadings for further examination (> 0·3) in PCA, it was loaded (0·279) on PC2.

Nutrients that were not associated with the same nutrients in PCA as in NR-FI indices included folate, Ca and vitamin B_6_. In the PCA, these nutrients were correlated with NR-FI_veg_ nutrients, although folate was originally included in NR-FI_carb_, and Ca and vitamin B_6_ in NR-FI_prot_. In addition, some nutrients that were included in two NR-FI indices showed correspondence only to one. For instance, potassium was not significantly linked with NR-FI_carb_-associated nutrients, and thiamine and Fe were not significantly linked with NR-FI_prot_-associated nutrients.

### Food grouping

**Fig. 2.**
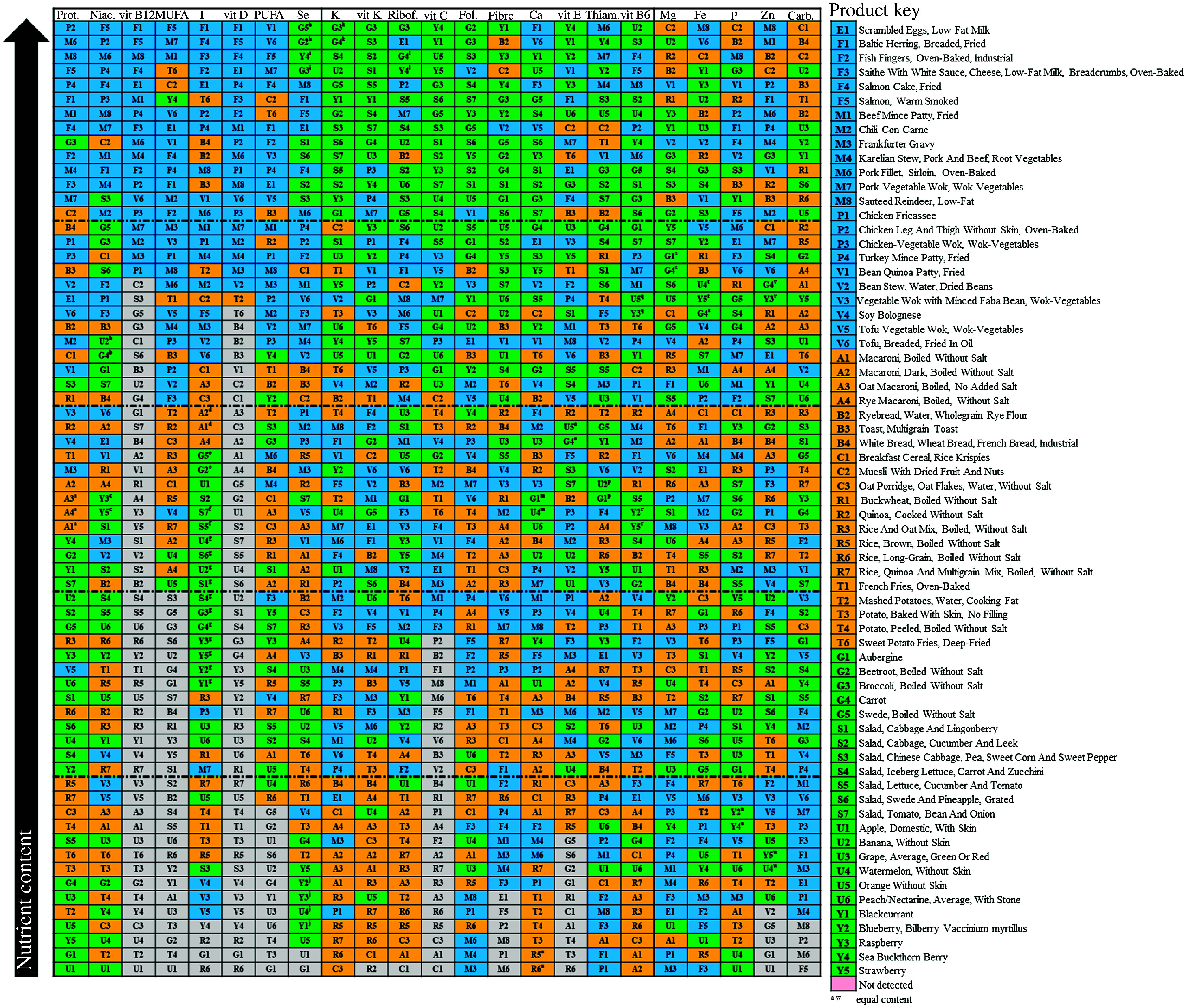
Food items sorted from highest to lowest based on the contents of the nutrients grouped in each principal component. The food item with the highest content is placed in the top cell and the food item with the lowest content is placed in the bottom cell. Protein group is in blue, carbohydrate group is in orange and fruit/vegetable group is in green. The broken lines indicate quintiles. If the nutrient content is zero (g, mg or µg), the food item is in grey.

The heat map shows that mainly nutrients that were most abundant in the protein foods were loaded on PC1, and that mostly nutrients that were at the highest level in fruits and vegetables were loaded on PC2 (Fig. 2). Foods that were categorised as carbohydrate sources were scattered among the components, but PC3 correlated positively with Mg, potassium and carbohydrate, three nutrients that were especially abundant in morning cereal products and breads.

**Fig. 3.**
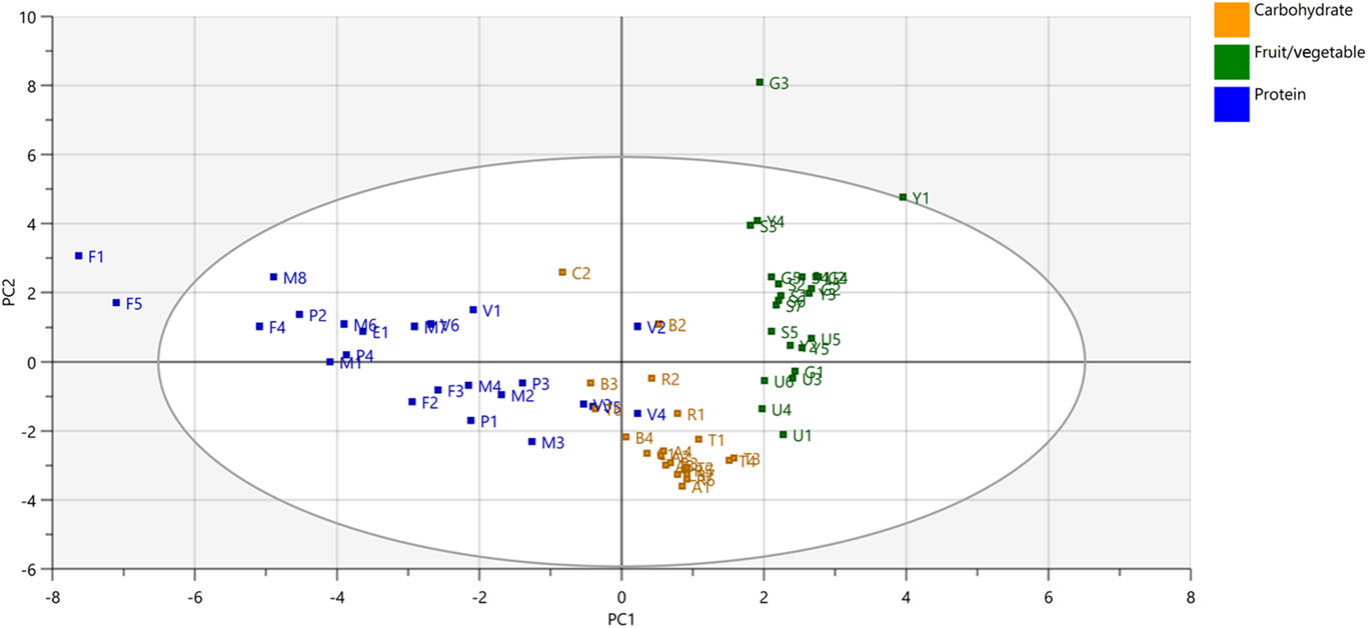
Score plots showing the grouping of protein sources, carbohydrate sources and fruits and vegetables based on their nutrient contents on principal components 1 and 2. Protein foods are in blue, carbohydrate foods are in yellow, and fruits and vegetables are in green. PC, principal component; E, egg dish; F, fish dish; M, meat dish; P, poultry dish; V, plant protein dish; A, pasta; B, bread; C, breakfast cereal; R, grain; T, tuber; G, greens; S, salad; U, fruit; Y, berry.

In the score plot, foods categorised as animal protein sources (eggs, fish, meat and poultry) were clustered together, while the fruit/vegetable group formed a separate cluster (Fig. 3, online Supplementary Fig. S1). Mixed plant protein stews containing vegetables (bean stew (V2), soy Bolognese (V4), minced faba bean and tofu wok dishes (V3 and V5)) were scored close to the carbohydrate group and also approached the fruit/vegetable group. Wheat and rice-based carbohydrate sources were closely clustered.

**Fig. 4.**
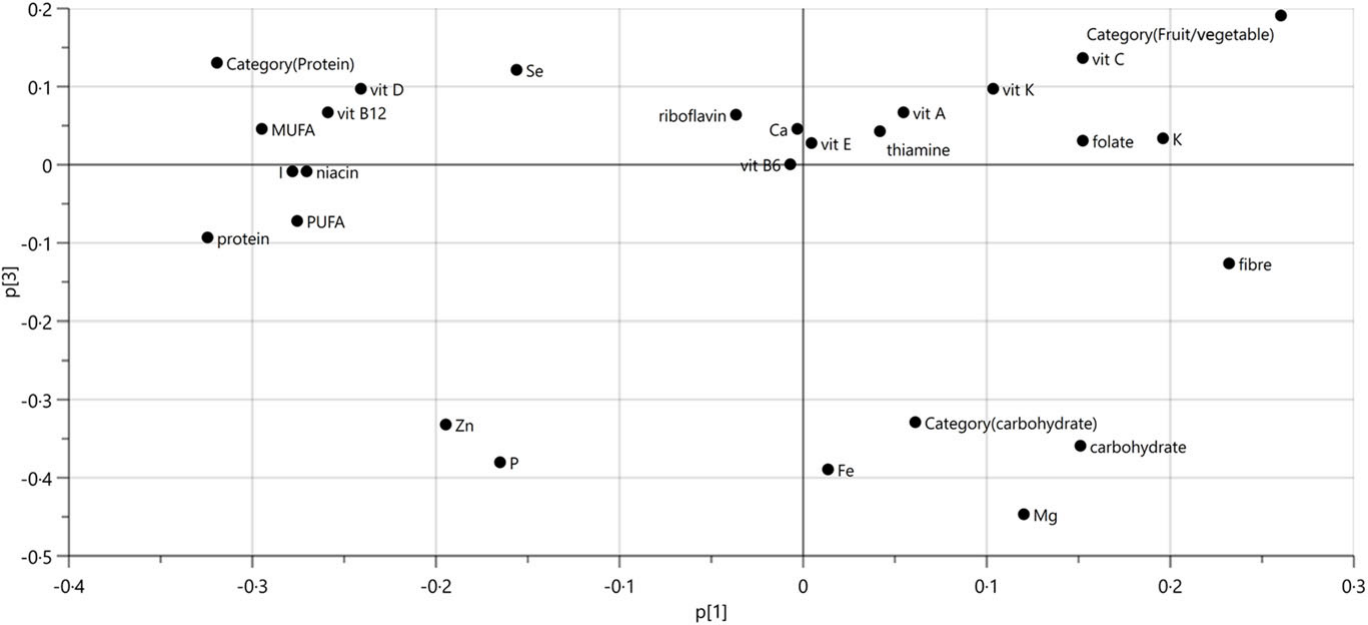
Loading plot showing the contribution of different nutrients in the grouping of protein, carbohydrate and fruit/vegetable foods on principal components 1 (p(1)) and 3 (p(3)).

Overall, the results were also demonstrated by the loading plot (Fig. 4), showing that PC3 could separate carbohydrate category foods together with carbohydrate, Fe and Mg, while protein category foods were strongly characterised by protein, niacin and vitamin B_12_ and the fruit/vegetable category by vitamin C, vitamin K and potassium.

## Discussion

Validation of nutrient index scores aims to ensure consistency with a measure of diet quality and should be implemented with science-driven tools^(4)^. Similar tools can also be applied to validate the accordance of product-group-specific nutrient indices with dietary guidelines and suggested nutritional functions. The validation process presented here produced sets of nutrients that largely correspond to the NR-FI baseline indices that were formed based on the national nutrient intake data^(14,15)^. This was especially true for the carbohydrate and fruit and vegetable indices, indicating carbohydrates to be major sources of Mg, Fe, phosphorous and available carbohydrate, and fruits and vegetables as major sources of potassium, vitamin K, vitamin C, fibre and thiamine. In correspondence to NR-FI_veg_, the major sources of vitamin A were vegetables. However, some differences were recorded between the intake-based indices and the sets of nutrients introduced in this paper. While our intake-based NR-FI indices highlight the role of carbohydrate sources, such as bread, as sources of folate, the plate model suggests higher intake of folate from fruits and vegetables. No carbohydrate foods made it to the highest quintile for folate sources. Furthermore, although potassium was included in the NR-FI_carb_ index, it did not show this association in the PCA and there were no carbohydrate sources in the highest quintile for potassium content. Bread and potatoes are highly consumed staple foods in the Finnish diet and therefore serve as important sources of folate and potassium, respectively. The PCA indicates, however, that based on dietary recommendations, vegetables should be favoured as a source of folate and also as a source of potassium.

For the protein index NR-FI_prot_, protein, niacin, vitamin B_12_ and Se were validated as being provided mainly by protein sources in the plate model. In contrast, Ca, thiamine and vitamin B_6_ were associated with the fruit/vegetable group and Fe with the carbohydrate group. As mentioned, thiamine was included also in the NR-FI_veg_ index and Fe in the NR-FI_carb_ index^(15)^. Nevertheless, based on the nutrient content ranking, one or two of the highest contents for Ca, thiamine and Fe were measured from a protein source, indicating the relevance of meat products in providing Fe and thiamine^(27)^ and fish and tofu in providing Ca^(28–30)^. Furthermore, Zn and riboflavin showed some correlation with NR-FI_prot_-associated nutrients in the PCA. However, plant protein sources used in this study seemed not to provide much Zn unless they contained some form of (pseudo)cereal grains, for example, quinoa or breadcrumbs.

Although it has been acknowledged that plants provide high levels of Ca and Zn, it is also well known that some antinutrients and fibre can bind mineral nutrients in plant matrices, and therefore, these nutrients are more bioavailable from animal sources^(31)^. In addition, non-haem Fe in, for example, legumes and cereals is notably less bioavailable in comparison with haem Fe from animals^(31)^. Previous literature suggested that bioavailability of Ca from plant sources averages 30 %^(32)^. We tested the PCA with Ca values corrected for bioavailability (results not shown) and noted that ranking of protein dishes as sources of Ca was improved after the correction, highlighting fish and egg dishes, in particular. The top quintile for Ca content still contained fruits (blackcurrant, orange and raspberry) and root and cruciferous vegetables. Although the single portion of 350 g is very high for blackcurrants alone, for example, it is possible to eat 350 g of a combination of different fruits, root vegetables and crucifers in one meal, making the fruit/vegetable group a major source of Ca. However, it is apparent that the evaluation of bioavailability cannot be handled using statistical models without further control exercised by nutrition experts.

An intrinsic characteristic of an average Finnish diet is the high consumption of milk and dairy products, which are naturally rich in Ca and often supplemented with vitamin D^(33)^. High consumption makes them also an important protein source in Finland^(16)^. Therefore, Ca was included in the NR-FI_prot_ index^(14)^. However, in the main meals of the day (lunch or dinner), milk and dairy products seldomly serve as sole or major protein sources. In the current study, it was determined that including milk-containing carbohydrate foods in the dataset drove separation of Ca and vitamin D to form their own principal component. At the same time, foods in the protein food category did not extensively contain dairy products, further loosening the link among protein, Ca and vitamin D. Instead, the interphase between plant-based protein foods and carbohydrate foods became even more indistinct. In the Finnish diet, cooking fats, fat spreads and milk as a meal drink provide most (ca. 70 %) vitamin D^(16)^. Because plant-based protein foods are often moderate sources of Ca but do not provide vitamin D, this supports the concept applied in NR-FI_prot_ that the protein food group as such is not pivotal for vitamin D intake. Further, a bit surprisingly, dairy products were not a major component in protein foods in the food database recipes but more so in a few products in the carbohydrate food group. It was concluded, however, that carbohydrate sources should not be categorically promoted as good sources of Ca and vitamin D. Instead, because milk is largely consumed as a drink in Finland^(34)^, both Ca and vitamin D could be deemed as important nutrients in the product group of beverages which was not included in this study.

It was reported that Canadian adults who get their protein mainly (≥ 75 %) from animal sources tend to receive significantly higher amounts of niacin and vitamin B_6_ from their diet in comparison with people consuming more (≥ 25 %) plant proteins^(35)^. However, individuals consuming mainly animal protein also ingest significantly more SFA and cholesterol and less dietary fibre^(35)^. Meanwhile, as the proportion of plant protein over animal protein in the diet increases, the odds of not fulfilling the daily recommendations for riboflavin and niacin intake increases, and also the intake of vitamin B_6_ is reduced^(35)^. As riboflavin and vitamin B_6_ are widely available from plant-based food sources, this suggests that the consumption of fruits and vegetables is generally at a low level in Western populations, even in groups consuming mainly plant proteins. Breads and cereal grains are important protein sources for those who favour plant-based foods^(35)^, but according to our ranking, the riboflavin and vitamin B_6_ levels in cereal grain products are not very high. Therefore, it would be advisable to direct consumers to receive riboflavin and vitamin B_6_ from green vegetables and fruits. Niacin, on the other hand, can be obtained from mildly processed cereal grains, nuts and legumes^(36,37)^, the cornerstones of plant-based proteins. Taken together, inclusion of niacin in the NR-FI_prot_ index was validated. However, due to the overall low levels of riboflavin and vitamin B_6_ in plant protein foods, it could be re-evaluated whether including these vitamins in a protein index creates a bias; instead, the role of fruits and vegetables as sources of riboflavin and vitamin B_6_ could be highlighted.

It was determined that for the plant-based protein sources some nutrients that are indicated to differentiate the protein foods from other food groups do not occur naturally in plant protein foods at high levels. These include vitamins B_12_ and D, but also nutrients that are added as supplements or during cooking. For example, MUFA, PUFA and iodine were the nutrients in PC1 that had plant protein sources in the highest quintile, yet cooking oil contributes to the MUFA and PUFA content and iodised salt contributes to the iodine content of plant-based patties and wok dishes. Furthermore, even a small amount of egg in an otherwise plant-based recipe contributes to the levels of vitamins B_12_ and D, as determined in the case of breaded tofu (V6). This makes the plant protein product group prone to variation caused by cooking habits and may artificially improve the ranking of plant protein sources within the protein index. This might also make adding salt or fat to plant protein dishes tempting. Thus, our decision to exclude MUFA, PUFA and iodine from the NR-FI_prot_ index is justified, although they could help to bring forward animal protein options with beneficial fatty acid profiles and natural iodine, as in fish, if included in the index.

It is notable that more than one plant protein food appeared in the highest quintile for Mg, Fe and phosphorous that were clustered in the carbohydrate source-related PC3. The observation that plant protein sources grouped close to carbohydrate foods relates also to a larger question of dietary shift and future roles for food groups in the plate model in providing nutrients^(1,38)^. Inclusion of Ca and Fe in the NR-FI_prot_ might help to spot the best plant protein sources for these nutrients, along with some important sources in omnivorous diets, that is, fish (especially with bones) and meat, respectively. It could be possible to develop a vegan nutrient index based on a modified plate model with a higher proportion of protein food and a lower proportion of carbohydrate food. This would potentially help to identify the best plant sources of Fe and Ca, but also of Zn and riboflavin, to ensure adequate intake and absorption^(39)^. Similarly, foods can be selected, and the portion sizes can be modified to correspond to any specific diet, dietary recommendation or food cultural context to form more relevant product-group-specific sets of nutrients. It is also possible to include nutrients to limit, although they are not recommended to be included in the nutrient indices used as nFU in nutritional LCA^(7)^.

In the context of NR-FI_prot_, it might be concluded that taking the protein food group as a whole, it has the potential to provide protein, Ca, Se, Fe, Zn, niacin and thiamine. Other sources might better secure vitamin B_6_ or riboflavin intake without the risk of increased intake of low-quality fats. In addition, although legumes and nuts are regarded as potentially good sources of Se, Se content in plants depends on soil Se levels, which can vary considerably^(40)^. Eggs and meat are the most important sources of thiamine in the Finnish diet^(16)^, but based on the current analysis, thiamine can also be obtained from various plant sources, including foods from all three categories. Therefore, it is apparent that both protein foods and the fruit/vegetable group are relevant sources of thiamine in the plate model. Albeit that vitamin B_12_ is almost solely provided by animal protein, it is an essential nutritional component and thus should be included in the protein index.

To summarise the results of the validation process, it is suggested that NR-FI_prot_ should contain protein, Ca, Fe, Se, Zn, vitamin B_12_, niacin, and thiamine, NR-FI_carb_ should contain carbohydrate, fibre, Fe, Mg, and phosphorous, and NR-FI_veg_ should contain fibre, potassium, thiamine, vitamin C, vitamin K, vitamin A, folate, vitamin B_6_, riboflavin, and Ca.

### Conclusions

This study largely validated the choice of nutrients for the NR-FI indices. The statistical analysis indicated that different food groups provide a distinct set of nutrients in an everyday diet. However, when the plate model was used as a basis for selecting the portion sizes for different food groups, the role of fruits and vegetables as sources of group B vitamins (except B_12_) and Ca, in particular, was featured more in comparison with the original indices. However, this is consistent with many dietary guidelines and suggests that nutrient indices as part of LCA can be used to encourage consumption of healthy, low-energy plant-based foods with typically low environmental impact. Although PCA is a convenient way to visualise correlations between different nutrients in foods, it requires expert interpretation and evaluation regarding bioavailability issues and overlapping of food groups in terms of nutrient compositions. Thus, selecting nutrients for nutrient indices using both statistical modelling and professional knowledge provides the best result.

## Supporting information

Kårlund et al. supplementary material Kårlund et al. supplementary material

## Supplementary material

For supplementary material/s referred to in this article, please visit https://doi.org/10.1017/S0007114524000709
